# The regulatory roles of dysregulated lactate dehydrogenase A in anti-tumor immunity within immune cells

**DOI:** 10.3389/fimmu.2026.1847542

**Published:** 2026-06-17

**Authors:** Xiaole Song, Shitong Lin, Qianwen Liu, Xuerou Chen, Yajuan Ma, Xiaoran Zhang, Fang Ren

**Affiliations:** 1 Department of Gynecologic Oncology, The First Affiliated Hospital of Zhengzhou University, Zhengzhou, Henan, China; 2 Department of Obstetrics and Gynecology, Union Hospital, Tongji Medical College, Huazhong University of Science and Technology, Wuhan, Hubei, China; 3 Cancer Biology Research Center (Key Laboratory of The Ministry of Education), Tongji Hospital, Tongji Medical College, Huazhong University of Science and Technology, Wuhan, Hubei, China

**Keywords:** anti-tumor immune response, lactate, lactate dehydrogenase A, lymphocytes, myeloid cell, macrophages

## Abstract

Malignant tumors maintain proliferative, invasive, and metastatic capacities through metabolic reprogramming. Lactate dehydrogenase A (LDHA), is an essential glycolytic enzyme that is aberrantly overexpressed in diverse human malignancies. The enzymatic activity of LDHA is tightly modulated by multiple post-translational modifications, including acetylation, phosphorylation, and lactylation. LDHA promotes the Warburg effect and lactate accumulation, while participating in tumor DNA damage repair, malignant progression, and drug resistance. LDHA and its metabolite lactate substantially modulate the differentiation and functional status of tumor-infiltrating immune cells, including lymphoid and myeloid cells. These metabolic changes remodel the tumor immune microenvironment and regulate anti-tumor immune responses in a context-dependent manner. This review systematically summarizes the regulatory mechanisms of LDHA in immune cell metabolism and anti-tumor immunity. Additionally, it discusses the clinical potential of LDHA as a promising anti-tumor therapeutic target and explores the application prospects of combining LDHA inhibitors with immunotherapies, such as chimeric antigen receptor T-cell (CAR-T) therapy and immune checkpoint blockade. This study aims to provide novel perspectives for the research and development of tumor targeted therapies.

## 1 Introduction

The sustained proliferation, invasion, and metastasis of malignant tumors are highly dependent on aberrant cellular metabolic reprogramming (1). The Warburg effect is the most representative metabolic hallmark of tumors, whereby tumor cells preferentially activate glycolysis and produce abundant lactate even under normoxic conditions. At present, the pivotal role of Lactate dehydrogenase A (LDHA)-mediated glycolysis in tumor progression has been extensively validated (2). However, existing reviews still reveal prominent knowledge gaps. Most studies focus on the oncogenic mechanisms of LDHA in tumor cells, yet ignore endogenous LDHA expressed in immune cells infiltrated in the tumor microenvironment (TME). Differential LDHA expression in distinct immune cells specifically regulates immune phenotypes, redox homeostasis, and biological functions. Immune cells do not merely adapt to TME passively; their intrinsic metabolic reprogramming serves as a critical internal factor determining immune activation or immunosuppressive differentiation. As a key rate-limiting enzyme of glycolysis, LDHA has been confirmed to modulate immune cell polarization, immune effector activation, and inflammatory phenotypic transformation.

A variety of immune cells, including macrophages, T lymphocytes, and myeloid cells, undergo LDHA-dependent metabolic remodeling in TME (3). Unlike tumor cells that primarily utilize glycolysis for rapid energy production, LDHA-mediated lactate production in immune cells not only directly regulates cell viability and immune functions, but also synergistically modulates reactive oxygen species homeostasis, antioxidant capability, glutamine metabolism and lipid synthesis pathways, ultimately polarizing immune cells toward pro-inflammatory, cytotoxic or immunosuppressive phenotypes. At present, most studies targeting LDHA focus on suppressing tumor cell proliferation, while limited literature has systematically summarized the differential expression profiles, metabolic regulatory networks and immunomodulatory mechanisms of LDHA across distinct immune cell subsets. This review focuses on the biological functions of LDHA in immune cells. We systematically summarize the expression patterns and metabolic regulatory mechanisms of LDHA in diverse tumor-infiltrating immune cells, and elucidate the intrinsic mechanisms by which it remodels the tumor immune microenvironment. This review aims to clarify the targeting advantages of LDHA in tumor immunometabolism, remedy the limitations of previous reviews, and provide theoretical basis for the establishment of LDHA-targeted combined strategies for tumor immunotherapy.

## 2 The structural localization and biological characteristics of LDHA

LDH is a tetrameric enzyme encoded by four distinct subunit genes, namely LDHA, LDHB, LDHC and LDHD. Each subunit has a molecular weight of approximately 35 kDa. Electrophoretic separation can identify five catalytically active LDH isoforms, consisting of two homomeric isoforms and three heteromeric isoforms. The canonical isoforms formed by LDHA (M subunit) and LDHB (H subunit) include LDH1 (four H subunits), LDH2 (three H subunits and one M subunit), LDH3 (two H subunits and two M subunits), LDH4 (one H subunit and three M subunits), and LDH5 (four M subunits). Among these isoforms, LDH1 is predominantly composed of LDHB, while LDH5 is mainly constituted by LDHA. Tissue distribution analyses reveal that LDHB is highly expressed in oxygen-rich aerobic tissues such as the myocardium and brain, whereas LDHA is largely enriched in glycolysis-prone tissues represented by white skeletal muscle (4–7).

**Figure 1:**
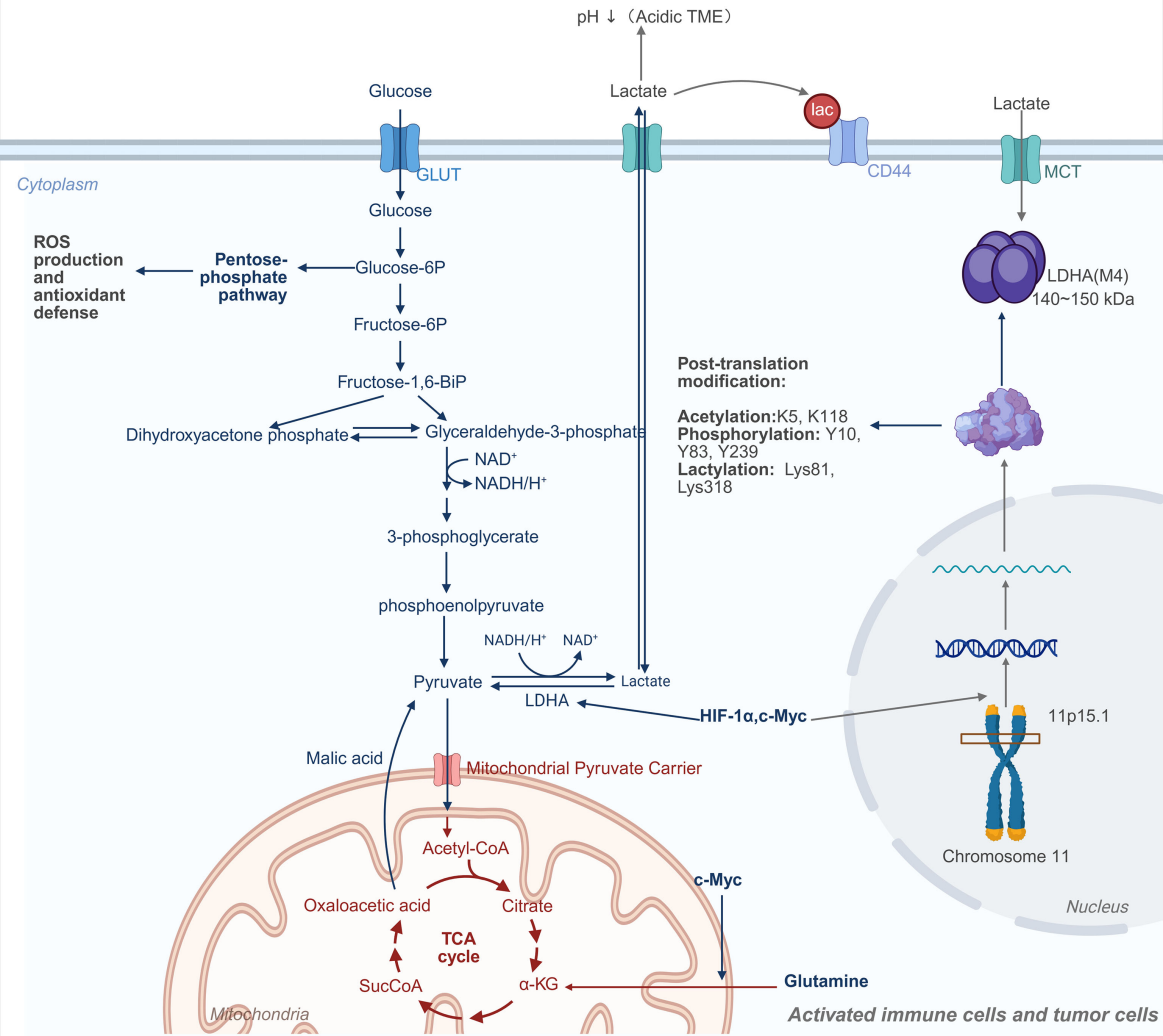
Schematic diagram of intracellular energy metabolism in tumor cells and activated immune cells. LDHA functions as a critical rate-limiting glycolytic enzyme that dominates intracellular energy metabolic reprogramming. Both tumor cells and activated immune cells exhibit enhanced glycolytic flux. Upregulated LDHA accelerates the conversion of pyruvate to lactate, sustains intracellular glycolysis, and alters energy allocation to meet the high bioenergetic demands of malignant proliferation and immune activation. This aberrant metabolic pattern affects intracellular signaling transduction and cellular biological behaviors. (Created in BioRender. song, X. (2026) https://BioRender.com/2v8q0l8).

In humans, the LDHA gene is mapped to chromosome 11p15.1 and encodes a 332-amino-acid polypeptide chain (**Figure 1**). The molecular weight of intact LDH holoenzyme ranges from 140 kDa to 150 kDa (6). All LDH subunits possess conserved catalytic domains, including active sites, proton acceptor sites, cofactor-binding domains and substrate-binding residues (8, 9). LDHA and LDHB exert opposite catalytic effects: LDHA promotes lactate generation during glycolysis, while LDHB accelerates lactate oxidation to produce pyruvate and reduced nicotinamide adenine dinucleotide (NADH), which further participates in the biosynthesis of glycogen, nucleotides and fatty acids.

In tumor tissues, LDHA-driven Warburg effect accelerates glucose consumption and induces massive lactate accumulation. Although glycolysis yields lower levels of adenosine triphosphate (ATP) than oxidative phosphorylation, its rapid metabolic rate sustains sufficient energy supply and provides biosynthetic substrates for malignant tumor progression. LDHA exhibits heterogeneous expression patterns and divergent metabolic regulatory functions across immune cell subsets. Activated T cells markedly upregulate LDHA to satisfy heightened bioenergetic requirements (10). Pro-inflammatory M1 macrophages depend on robust glycolysis alongside mitochondrial dysfunction, whereas anti-inflammatory M2 macrophages prefer mitochondrial oxidative phosphorylation. In naive B cells, LDHA serves as a vital intrinsic metabolic modulator to sustain glycolytic flux and ATP generation. During terminal differentiation into plasma cells, B cells undergo metabolic reprogramming characterized by the repression of LDHA-driven glycolysis and enhanced tricarboxylic acid (TCA) cycle activity (11, 12). Collectively, these heterogeneous traits determine the disparate biological functions of LDHA in distinct immune populations.

Beyond glycolysis, intracellular metabolic networks are highly interconnected; c-Myc-mediated glutaminolysis converts glutamine into α-ketoglutarate (α-KG) to maintain the homeostasis of the TCA cycle. Malate derived from the TCA cycle is transported into the cytoplasm and catalyzed into pyruvate by malic enzyme, which continuously provides precursors for LDHA-dependent lactate synthesis (13, 14). Apart from its canonical role in energy metabolism, LDHA-mediated lactate production is reported to modulate the lactylation status of DNA repair proteins, including flap structure-specific endonuclease 1 (FEN1), X-ray repair cross-complementing protein 5 (XRCC5), and X-ray repair cross-complementing protein 6 (XRCC6), specifically in lung adenocarcinoma (LUAD). Such lactylation remodeling may help maintain the functional stability of these repair proteins and thereby support the normal activity of the non-homologous end joining (NHEJ) pathway in this defined tumor context (15). The transcription factor FoxO3a suppresses glycolysis and the pentose phosphate pathway to downregulate LDHA activity, which reduces the production of NADPH and reactive oxygen species (ROS), impedes mitochondrial metabolism, and ultimately reverses the drug-resistant phenotype of breast cancer (16). Collectively, LDHA-mediated metabolic reprogramming markedly modulates the crosstalk between tumor cells and immune cells, ultimately remodeling an immunosuppressive tumor microenvironment.

The enzymatic activity of LDHA is precisely regulated by multiple post-translational modifications (PTMs), among which acetylation, phosphorylation, and lactylation serve as the predominant regulatory patterns. Acetylation primarily occurs at the K5 and K118 lysine residues of LDHA (17, 18). With respect to phosphorylation regulation, tyrosine kinases, including human epidermal growth factor receptor 2 (HER2), fibroblast growth factor receptor 1 (FGFR1), and proto-oncogene tyrosine-protein kinase spare respiratory capacity (SRC), mediate the phosphorylation of LDHA at Y10 and Y83 residues, which constitutively activates LDHA and accelerates malignant tumor progression (19–21). Additionally, Stress-activated mitogen-activated protein kinase-interacting protein 1 (SIN1) recruits threonine tyrosine kinase (TTK) to induce LDHA phosphorylation at the Y239 residue, thereby potentiating glycolysis and lactate accumulation in tumor cells. Accumulated lactate upregulates histone H3 lysine 18 (H3K18) lactylation and facilitates glucose transporter 3 (GLUT3) transcription, forming a positive feedback loop of glucose metabolism that is correlated with adverse patient prognosis (22). Meanwhile, non-coding RNAs (ncRNAs) are also implicated in the phosphorylation modulation of LDHA. CircTATDN3 can persistently activate the glycolytic pathway in colon cancer cells by modulating LDHA phosphorylation, thereby driving tumor metabolic reprogramming (23). With regard to tumoral lactylation, alanyl-tRNA synthetase 1 (AARS1) catalyzes the lactylation of LDHA at the K81 and K318 residues to enhance the enzymatic activity of LDHA and augment the Warburg effect. Loss of LDHA lactylation induces the accumulation of glycolytic intermediates, reduced ATP production, and impaired DNA repair capacity in tumor cells (15). Collectively, as a pivotal rate-limiting enzyme in glycolysis, LDHA modulates tumor progression via multiple regulatory pathways, including post-translational modification, DNA damage repair, and transcriptional regulation, making it a promising target for clinical translation in anti-tumor therapy and drug resistance reversal.

### 2.1 LDHA modulates the effects of anti-tumor activity in immune cells

LDHA in immune cells modulates the activation, differentiation, and migration of immune cells within TME. It governs the secretion of key cytokines, including interferon-γ (IFN-γ), tumor necrosis factor-α (TNF-α), and granulocyte colony-stimulating factor (G-CSF), which alters immune cytotoxicity and further reshapes anti-tumor immune homeostasis.

## 3 Mechanisms of LDHA in regulating lymphocyte-mediated tumor immunity

**Figure 2:**
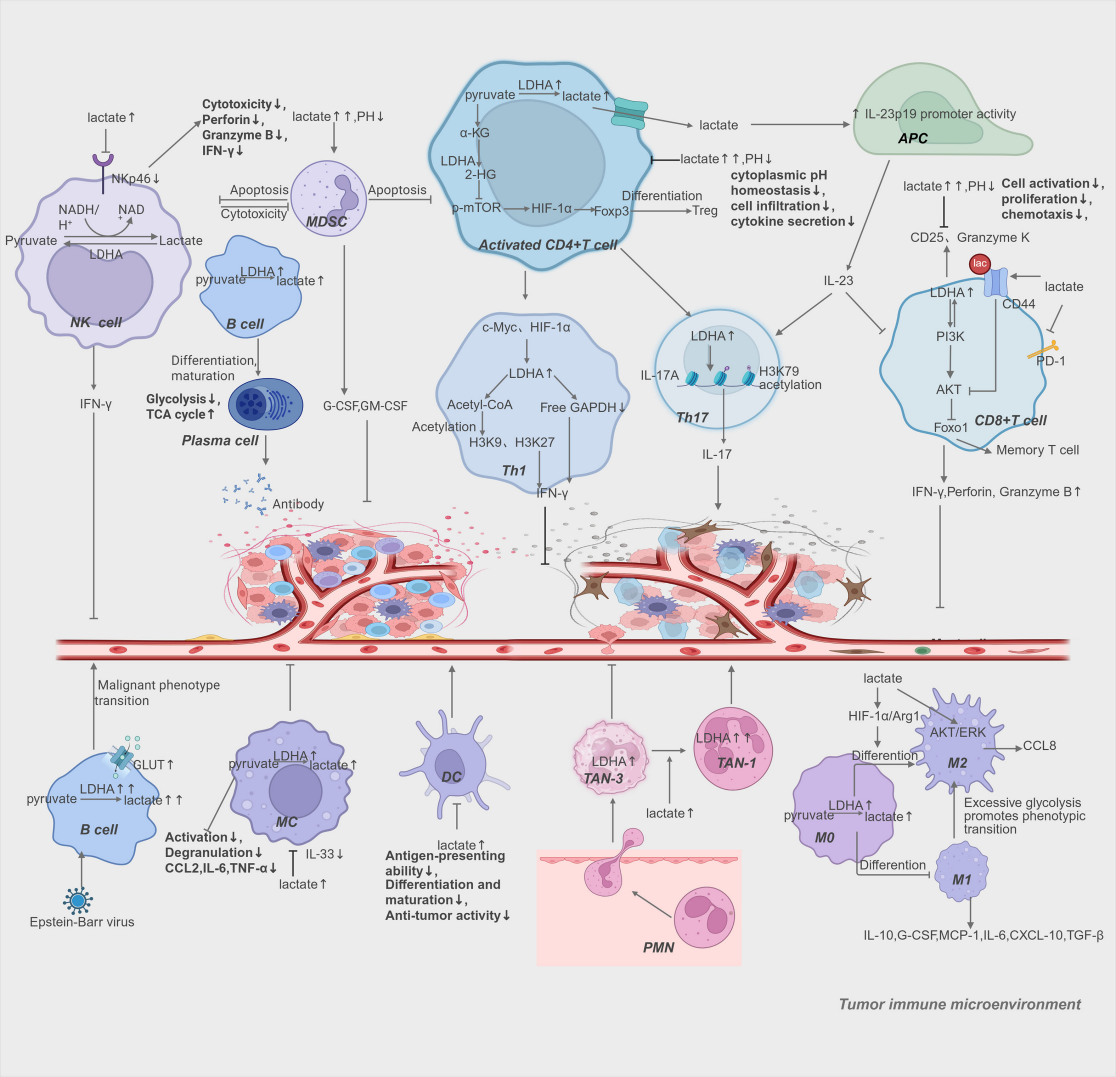
Regulation of the tumor immune microenvironment by the LDHA/lactate axis. This figure illustrates the central regulatory role of LDHA and its metabolic product, lactate, in the tumor immune microenvironment. The central area represents the tumor tissue. The upper and lower areas depict various immune cells infiltrating the tumor surroundings. The LDHA/lactate axis differentially modulates the function of these immune cells. On one hand, it can intrinsically regulate immune cell activation, energy metabolism, and cytokine production. On the other hand, lactate released into the extracellular tumor microenvironment acts by suppressing receptor expression (e.g., NKp46 on NK cells), driving epigenetic reprogramming, and influencing the internal molecular mechanisms of immune cells, collectively reshaping the immunosuppressive landscape. (Created in BioRender. song, X. (2026) https://BioRender.com/2v8q0l8).

LDHA expression is markedly upregulated in tumor-infiltrating lymphocytes, including T lymphocytes, B lymphocytes, and natural killer (NK) cells, within tumor tissues and TME. Acting through multiple regulatory cascades, LDHA modulates the biological functions of these infiltrating lymphocytes, thereby altering their regulatory effects on tumor progression (**Figure 2**). In this section, we systematically elaborate the core regulatory modes of LDHA in three major types of tumor-infiltrating lymphocytes.

### 3.1 CD4+ T cells

We categorize the regulatory mechanisms herein into four independent modules: intrinsic LDHA metabolic regulation, extracellular lactate bioactivity, protein lactylation, and TME acidosis-associated immune suppression.

Activated cluster of CD4^+^ T cells undergo a metabolic switch from oxidative phosphorylation to aerobic glycolysis, which relies heavily on LDHA. Within CD4^+^ T cells, LDHA catalyzes pyruvate conversion to lactate and regenerates nicotinamide adenine dinucleotide (NAD^+^), maintaining steady glycolytic flux and sufficient ATP supply to support T cell activation (10). Such intrinsic metabolic properties of LDHA are critical for maintaining intracellular redox homeostasis and supporting robust effector immune responses in T cells.

Lactate accumulates inside and outside T cells and exerts independent regulatory effects beyond LDHA enzymatic activity. Intracellular lactate buildup restricts CD4^+^ T proliferation and activation. Excessive extracellular lactate saturates monocarboxylate transporter 1 (MCT1), impairs T cell metabolic fitness, disrupts cytoplasmic pH homeostasis, and weakens tumor infiltration and cytokine secretion (24, 25). Collectively, activated T cells rely on LDHA-driven glycolysis to sustain immune activation and effector functions, whereas lactate accumulation induces concentration-dependent inhibitory effects, thereby adversely disrupting T-cell immune potency.

Beyond lactate-related biological effects, lactate-induced extracellular acidification represents an independent immunosuppressive pattern. Acidic conditions alter T cell surface receptor profiles and inhibit cytolytic granule release. Tumor cell-specific LDHA deletion reduces lactate secretion and microenvironmental acidification, downregulates Hypoxia-inducible factor 1α (HIF-1α) and vascular endothelial growth factor-A (VEGF-A), and increases CD3^+^/CD4^+^ T cell infiltration to restrain tumor progression (26, 27).

LDHA-mediated metabolic reprogramming varies markedly among CD4^+^ T subsets, including T helper 1 (Th1), T helper 17 (Th17), regulatory T (Treg), T helper 2 (Th2), and T follicular helper (Tfh) cells (28). Such metabolic heterogeneity determines cell differentiation fate and adaptive anti-tumor immunity. Overall, LDHA-dependent glycolysis is indispensable for T cell activation; by contrast, excessive extracellular lactate and subsequent acidification exert context-dependent inhibitory effects, explaining inconsistent immune outcomes across different tumor settings.

#### 3.1.1 Th1 cells

This subsection centers on intrinsic LDHA-governed metabolism and epigenetic modulation in Th1 cells. In Th1 cells, LDHA controls IFN-γ expression through two coordinated yet separate routes: post-transcriptional de-repression and epigenetic activation.

In the post-transcriptional cascade, enhanced LDHA-dependent glycolysis sequesters glyceraldehyde-3-phosphate dehydrogenase (GAPDH). This reduces the pool of free GAPDH and weakens its binding to the AU-rich element of IFN-γ mRNA, ultimately alleviating translational repression. During T cell activation, the HIF-1α and c-Myc signaling pathways collectively upregulate LDHA. Increased glycolytic flux produces abundant acetyl-CoA, which elevates histone H3 lysine 9 (H3K9) and H3K27 acetylation at the IFN-γ promoter. This remodels chromatin accessibility and recruits RNA polymerase II to drive gene transcription. Notably, this epigenetic regulatory pathway functions independently of GAPDH-mediated post-transcriptional control (10, 29).

Overall, LDHA supports Th1 anti-tumor activity by boosting IFN-γ production through multiple regulatory layers.

#### 3.1.2 Th17 cells

Within Th17 cells, LDHA orchestrates intracellular glycolytic programming and also influences immune behavior via lactate-driven extracellular actions.

LDHA stimulates interleukin-17A (IL-17A) protein expression via epigenetic mechanisms, specifically by increasing histone H3 lysine 79 acetylation at the IL17A gene locus to facilitate Th17 activation (10). Additionally, LDHA sustains anaerobic glycolysis in Th17 cells to maintain intracellular redox homeostasis and ATP production, while facilitating the activation of the phosphoinositide 3-kinase (PI3K)/protein kinase B (AKT) signaling axis through the conversion of phosphatidylinositol-4, 5-bisphosphate to phosphatidylinositol-3, 4, 5-trisphosphate (30). Activated AKT directly enhances glucose uptake and phosphorylation by promoting GLUT1 membrane translocation and activating hexokinase 2 (HK2). Moreover, AKT phosphorylates and inhibits glycogen synthase kinase 3 (GSK3) and other glycolytic negative regulators, thereby further amplifying glycolytic flux. In addition, AKT-mediated phosphorylation inactivates the transcriptional repressor forkhead box O1 (FoxO1), forming a positive feedback loop that persistently upregulates LDHA expression (31, 32).

Metabolically derived lactate produced by LDHA is exported into TME, where it acts as an extracellular signaling molecule independent of LDHA enzymatic activity. Elevated extracellular lactate enhances toll-like receptor (TLR)-triggered IL-23p19 promoter activity in antigen-presenting cells, thereby increasing IL-23 secretion (33). This cytokine restricts the infiltration of CD8^+^ T cells and promotes IL-17 secretion by peptide-activated CD4^+^ T helper cells (34). Furthermore, lactate specifically suppresses IFN-γ production in Th17 cells, polarizing the immune response toward a proinflammatory Th17 phenotype (33). In summary, LDHA and its downstream metabolite lactate exert distinct but interconnected functions in Th17-dependent tumor immunity. Within Th17 cells, LDHA sustains glycolytic flux and drives Th17 activation and IL-17A production; in the TME, extracellular lactate serves as an independent immunomodulatory mediator to trigger IL-23 production, facilitate Th17 polarization, and inhibit IFN-γ secretion.

#### 3.1.3 Treg cells

In Treg cells, LDHA reshapes metabolic profiles and drives histone lactylation-related epigenetic reprogramming to govern Th17/Treg homeostasis (35).

LDHA facilitates the conversion of α-ketoglutarate to 2-hydroxyglutarate, thereby inhibiting mammalian target of rapamycin (mTOR) phosphorylation and downstream HIF-1α generation. Lower HIF-1α abundance stabilizes forkhead box protein P3 (FOXP3), tipping cell differentiation toward the Treg lineage and fostering an immunosuppressive tumor microenvironment (36).

Furthermore, deubiquitination modification enhances LDHA protein stability and aggravates intracellular lactate accumulation. Excessive lactate potentiates histone lactylation, which transcriptionally activates ATP citrate lyase (ACLY), triggers fatty acid synthesis, and facilitates Treg phenotypic transformation, ultimately driving malignant tumor progression (35). Collectively, the regulatory effects of the LDHA/lactate axis on Th17/Treg differentiation are tightly associated with the intrinsic transcriptional characteristics of immune cells. Targeting lactate-dependent immunosuppressive pathways exhibits promising therapeutic potential for tumor treatment. Pharmacological interventions, including the inhibition of MCT1 using AZD3965, direct suppression of LDHA activity with GSK2837808A, and the blockade of NADH production by rotenone, can effectively rebalance Th17/Treg homeostasis and ameliorate the immunosuppressive state of the tumor microenvironment (37).

#### 3.1.4 Th2 and Tfh cells

The regulatory roles of LDHA in Th2 and Tfh cells remain incompletely characterized, with most evidence derived from correlational and metabolic studies.

In Th2 cells, extracellular lactate-related biological effects are closely linked to cell polarization. A positive correlation between MCT4 expression and Th2 cell infiltration has been identified in lung adenocarcinoma. Considering the essential role of MCT4 in exporting LDHA-derived lactate, the LDHA-lactate axis is speculated to facilitate Th2 polarization (38). In cutaneous T-cell lymphoma, elevated LDHA expression upregulates IL-25, which in turn activates the signal transducer and activator of transcription 3 (STAT3) cascade and promotes IL-13 secretion. This feedback favors the formation of a Th2-skewed immunosuppressive microenvironment and accelerates tumor progression (39, 40).

In Tfh cells, functional alterations are mainly driven by extracellular lactate-mediated metabolic perturbation. Multiple studies have demonstrated that LDHA overexpression induces glucose depletion and lactate accumulation within tumor tissues, accompanied by markedly decreased Tfh cell infiltration (41, 42). At present, research regarding LDHA- and lactate-mediated regulation of Tfh cells is still in its infancy, and many underlying mechanisms remain largely unexplored.

### 3.2 CD8+ T cells

#### 3.2.1 Cell-Intrinsic metabolic functions of LDHA

LDHA functions as a central metabolic regulator within CD8^+^ T cells, governing glycolytic reprogramming and cell fate differentiation independent of extracellular lactate effects.

In CD8^+^ T cells, LDHA acts as a core metabolic regulator governing glycolytic reprogramming and cell differentiation. LDHA-driven glycolysis activates the PI3K-AKT-FoxO1 axis to induce FoxO1 phosphorylation and inactivation, which promotes effector T cell differentiation but constrains memory T cell formation (43, 44). Activated PI3K signaling further upregulates LDHA via c-Myc-dependent pathways, and enhances glutaminolysis to supply glycolytic substrates, forming a positive feedback loop. This metabolic reprogramming satisfies the biosynthetic demands of activated CD8^+^ T cells and sustains the production of perforin, granzyme B and effector cytokines (43).

#### 3.2.2 Extracellular regulatory modes of lactate

In the TME, lactate exerts regulatory roles through two distinct modes: non-enzymatic metabolic modulation and AARS1-dependent protein lactylation, rather than relying on LDHA activity or pH variation.

Tumor-derived lactate triggers AARS1-dependent extracellular lactylation of cluster of CD44 on CD8^+^ T cells, which disrupts CD44–hyaluronic acid binding and inhibits downstream AKT activation, thereby impairing anti-tumor immune responses (45). Lactate also induces AARS1-catalyzed programmed death ligand 1 (PD-L1) lactylation at the K280 residue. This modification blocks HUWE1-mediated ubiquitination and degradation, leading to PD-L1 accumulation and tumor immune escape (46).

Extracellular lactate differentially modulates programmed death 1 (PD-1) expression across immune cell subsets, particularly in highly glycolytic tumors including MYC-amplified liver tumors, colorectal cancer (CRC), and LUAD. In such tumor-specific contexts, lactate absorbed via MCT1 upregulates PD-1 expression in regulatory T cells while dampening PD-1 levels in effector CD8^+^ T cells (47). Beyond pH-dependent regulation, lactate accumulation in such defined tumor microenvironments is capable of compromising the cytotoxic activity and tumor infiltration capacity of CD8^+^ T cells. As documented in CRC and LUAD experimental models, lactate may engage endothelial cell-specific molecule 1 (ESM1) to modulate AKT1 signaling, which is accompanied by the attenuation of DNA damage response (DDR) and cGAS-STING pathway activity, thereby limiting CD8^+^ T cell tumor infiltration (48, 49). Notably, these mechanistic associations are context-dependent and cannot be generalized to all tumor types or immune cell settings. Collectively, identical lactate metabolic signals trigger disparate biological outcomes in distinct immune cells due to intrinsic differences in intracellular signaling networks. This discrepancy is markedly evident between Treg cells and CD8^+^ T cells, which fundamentally drives metabolic heterogeneity and immunosuppression within the tumor microenvironment.

### 3.3 NK cells

#### 3.3.1 Cell-intrinsic role of LDHA in NK cell function

Within NK cells, LDHA acts as a pivotal metabolic regulator to sustain stable glycolytic flux and support energy-intensive effector biological processes (50). Mechanistically, LDHA catalyzes the reduction of pyruvate to lactate, thereby regenerating NAD^+^ to sustain continuous glycolysis and ATP production. This metabolic process provides energy substrates for cytokine synthesis and cytotoxic granule exocytosis. Notably, IFN-γ secretion by NK cells displays stringent reliance on LDHA enzymatic activity, highlighting the indispensable function of LDHA-driven glycolysis in maintaining NK cell effector capacity (50, 51).

#### 3.3.2 Extracellular lactate-mediated actions and protein lactylation

Extracellular lactate acts through two mechanistically independent pathways to shape NK cell function: receptor-mediated biological regulation and lysine lactylation as a post-translational modification. These two modes are closely interconnected but operate via distinct molecular mechanisms.

Distinct from the cell-intrinsic metabolic properties of LDHA, extracellular lactate accumulated within TME exerts potent immunosuppressive effects via specific receptor-dependent signaling cascades. Lactate selectively downregulates the expression of natural killer cell p46-related protein (NKp46) in NK cells, while exerting negligible suppressive impacts on natural killer group 2 member D (NKG2D) and natural killer cell p30-related protein (NKp30), indicating a receptor-specific regulatory pattern of lactate in modulating NK cell activity (52). Such targeted inhibition impairs NK cell cytotoxicity, reduces the secretion of perforin and granzyme B, and represses IFN-γ production. NK cells are more susceptible to lactate-induced functional impairment than CD8^+^ T cells, reflecting inherent metabolic heterogeneity across immune populations.

Preclinical evidence has confirmed that pharmacological inhibition of LDHA combined with PD-1 blockade effectively restricts tumor growth and improves survival by restoring NK cell activity (53). Recent mechanistic investigations have demonstrated that lactate-induced elevation of lysine lactylation is tightly correlated with NAD^+^ metabolic perturbation and mitochondrial fragmentation, which substantially compromises the anti-tumor capacity of NK cells. Supplementation with the NAD^+^ precursor nicotinamide riboside and the sirtuin 3 (SIRT3) activator HKL activates deacylase SIRT3, decreases the lactylation levels of mitochondrial function- and oxidative stress-associated proteins, including Rho-associated kinase 1 (ROCK1), enolase 1 (ENO1), and phosphoglycerate kinase 1 (PGK1). This intervention ameliorates metabolic dysfunction, preserves mitochondrial structural integrity, and ultimately restores the cytotoxic activity of NK cells (54).

#### 3.3.3 Acidic microenvironment and immunosuppressive network effects

Lactate release driven by LDHA induces TME acidification, which independently suppresses NK cell activity and reshapes the immune landscape. An acidic TME facilitates the accumulation of myeloid-derived suppressor cells (MDSCs) and simultaneously induces apoptotic cell death in T cells and NK cells, thereby constructing an intricate immunosuppressive network that disrupts intrinsic immune surveillance (55). In LDHA-deficient tumor cells, diminished lactate secretion alleviates microenvironmental immunosuppression, enhances NK cell-dependent cytotoxicity, and impedes MDSC infiltration, collectively strengthening tumor immune control (56). These divergent yet complementary mechanisms emphasize the necessity of multi-targeted strategies targeting lactate metabolism. Therapeutic approaches focusing on modulating intracellular LDHA activity in immune cells, blocking extracellular lactate-dependent signaling, or neutralizing microenvironmental acidity can synergistically potentiate NK cell cytotoxicity and efficiently constrain malignant tumor progression.

### 3.4 B cells

#### 3.4.1 Cell-intrinsic functions of LDHA in B cell immunity

Distinct from CD4^+^ T cells, B cells exhibit unique metabolic plasticity under physiological conditions; resting B cells undergo a metabolic transition from glycolysis to oxidative phosphorylation to facilitate plasma cell differentiation and antibody affinity maturation (11, 12).

Epstein-Barr virus (EBV) infection profoundly remodels the intrinsic metabolic landscape of B cells. EBV upregulates MYC and HIF-1α expression, which synergistically enhances the transcription and enzymatic activity of LDHA in infected B cells. Such cell-autonomous metabolic reprogramming elevates intracellular glycolytic flux, increases pyruvate availability for LDHA-mediated catalysis, and promotes lactate accumulation. Concurrently, EBV infection enhances glucose uptake and upregulates GLUT1 expression, while repressing the transcriptional levels of TCA cycle and oxidative phosphorylation-related genes. This metabolic alteration diminishes mitochondrial respiration and establishes a typical Warburg phenotype, thereby supporting malignant transformation and uncontrolled B cell proliferation (57, 58).

In diffuse large B-cell lymphoma, LDHA knockdown markedly reduces lactate production, elevates the NAD^+^/NADH ratio, and decreases ROS accumulation. Mechanistically, suppressed LDHA expression inhibits the phosphorylation of STAT5, thereby restraining lymphoma cell proliferation and migration and triggering cellular apoptosis. Reduced ROS abundance further blocks ROS-mediated HIF-1α upregulation, confirming the regulatory role of LDHA in modulating cellular hydrogen peroxide generation and oxidative stress homeostasis (59, 60).

#### 3.4.2 Extracellular lactate-mediated immunomodulation

Lactate derived from LDHA-dependent glycolysis is actively exported from EBV-infected B cells via MCTs, generating a lactate-rich extracellular microenvironment with independent immunomodulatory properties. Excessive extracellular lactate exceeds the metabolic clearance capacity of adjacent immune cells and induces B cell exhaustion through receptor-dependent signaling cascades. Furthermore, lactate accumulation facilitates the expansion and activation of MDSCs with potent immunosuppressive functions, ultimately constructing a tumor-permissive immune microenvironment (61).

#### 3.4.3 Acidic microenvironment effects

Lactate efflux from metabolically reprogrammed B cells drives extracellular acidification, forming an acidic TME with broad immunosuppressive impacts. Acidic stress further dysregulates adhesion molecule expression and induces malignant morphological alterations in B cells. Similar acidic-dependent malignant alterations have also been observed in EBV-associated malignancies, including oral squamous cell carcinoma (62).

## 4 Mechanisms of LDHA in regulating myeloid cell tumor immunity

Beyond its regulatory roles in lymphocytes, LDHA also exerts widespread and heterogeneous immunomodulatory effects on myeloid cells. In this section, we systematically dissect the distinct regulatory patterns of LDHA and lactate across different myeloid cell subsets. Specifically, LDHA modulates the immune responses of multiple myeloid cell populations, including monocytes, macrophages, dendritic cells (DCs), tumor-associated neutrophils (TANs), MDSCs and mast cells (MCs).

### 4.1 Monocytes

Lactate inhibits monocyte migration and promotes tumor growth, invasion, and immune escape in a dose-dependent manner. Monocytes uptake extracellular lactate via MCT1, regardless of activation status. This lactate uptake inhibits LDHA-mediated glycolysis, depletes intracellular ATP, impairs phagocytic function, and ultimately facilitates immune evasion (63).

### 4.2 Macrophages

Tumor-associated macrophages (TAMs) exhibit high functional plasticity along the M1–polarization spectrum. This dynamic phenotypic transition is coordinately shaped by cell-intrinsic LDHA activity, lactate-mediated metabolic rewiring, and lactate-driven TME acidification, as well as lactate-associated histone lactylation modification. Mechanistically, tumor cell-intrinsic LDHA activates the extracellular signal-regulated kinase/yes-associated protein 1 (YAP1)/STAT3 transcriptional cascade to upregulate C-C motif chemokine ligand 2 (CCL2) and CCL7 expression, thereby facilitating TAM recruitment into tumor lesions. After infiltration, TAMs reciprocally secrete LDHA-enriched extracellular vesicles, which are internalized by tumor cells to reactivate the extracellular signal-regulated kinase (ERK)/YAP1/STAT3-CCL2/CCL7 axis and establish a bidirectional metabolic symbiosis between tumor cells and macrophages. Meanwhile, lactate accumulation within the TME stimulates macrophage-derived IL-23 production, which further activates Th17 cells to secrete IL-17 and amplifies the pro-tumorigenic cytokine network (64–66).

#### 4.2.1 Regulatory mechanisms of M1 macrophages​​

Mechanistically, M1 macrophage polarization and function are governed by cell-intrinsic LDHA-dependent metabolic reprogramming and lactate-initiated histone lactylation modification.

In M1 macrophages, LDHA sustains glycolytic flux and maintains redox homeostasis to support pro-inflammatory activation. Nevertheless, hyperactivated LDHA paradoxically suppresses M1 polarization. This paradoxical regulatory pattern is attributed to metabolic rewiring: despite glycolysis serving as the dominant metabolic signature of M1 macrophages, excessive glycolytic activity drives an M1-to-M2 phenotypic transition via lactate-mediated histone lactylation under lipopolysaccharide/IFN-γ stimulation (67). LDHA-driven glycolytic reprogramming reshapes the cytokine secretion profile of M1 macrophages, inhibiting anti-inflammatory mediators including IL-10, G-CSF, and monocyte chemoattractant protein-1 (MCP-1), while promoting the expression of pro-inflammatory cytokines such as IL-6, CXCL10, and transforming growth factor-β (TGF-β) (64). In glioblastoma, intrinsic LDHA activity represses brain and muscle ARNT-like 1 (BMAL1)-dependent transcriptional programs that govern M1 polarization and angiogenesis. Pharmacological blockade of downstream glycolytic pathways diminishes BMAL1 expression and glycolytic flux, sensitizing glioma cells to bevacizumab and restraining tumor progression (68).

#### 4.2.2 Driving mechanisms of M2 macrophages​​

M2 macrophage polarization is orchestrated through three interrelated layers: cell-intrinsic LDHA metabolic reprogramming, lactate-driven epigenetic modification, and TME acidosis-mediated immune regulation.

Elevated LDHA expression within macrophages boosts glycolytic flux to meet the bioenergetic demands of M2 differentiation, driving phenotypic polarization and remodeling the TME to accelerate breast cancer progression (69). Within the TME, accumulated lactate activates the AKT/ERK signaling cascade to trigger M2 polarization and CCL8 secretion, thereby promoting colorectal cancer metastasis through the CCL8/C-C chemokine receptor 5 (CCR5)/mammalian target of rapamycin complex 1 (mTORC1) axis (70).

Functioning as an epigenetic substrate, lactate induces histone H3 lysine 18 lactylation (H3K18la), which directly modulates transcriptional profiles to reinforce M2 polarization and dampen CD8^+^ T cell immune activity (71, 72). A positive feedback loop further amplifies this immunosuppressive phenotype: lactate upregulates LDHA expression via the pituitary tumor-transforming gene 3 pseudogene-mediated HIF-1α activation, thereby sustaining M2 polarization (73).

Beyond metabolic and epigenetic regulation, LDHA-fueled lactate release induces extracellular acidification. This acidic niche independently activates the HIF-1α/arginase 1 (ARG1) axis in a pH-dependent manner, further reinforcing M2 polarization (74–76).

Collectively, LDHA and lactate dynamically shape the pro-tumor TME and modulate malignant progression. The dichotomous effects of lactate on macrophage subsets stem from their inherent metabolic heterogeneity: pro-inflammatory M1 macrophages rely on robust glycolysis with limited mitochondrial activity, whereas anti-inflammatory M2 macrophages predominantly depend on mitochondrial oxidative phosphorylation (OXPHOS). MCTs mediate lactate transportation and are indispensable for M2-associated gene transcription. Within the acidic TME, extracellular lactate is converted into pyruvate and subsequently transported into mitochondria to fuel the TCA cycle, ultimately driving TAM polarization toward an immunosuppressive M2-like phenotype.

### 4.3 DCs

DCs serve as pivotal modulators of adaptive immunity; nevertheless, DCs within TME frequently facilitate immunosuppression and malignant tumor progression. The functional impairment of DCs is mainly driven by extracellular lactate-mediated biological effects and TME acidosis-related immune alteration, with additional contributions from intrinsic metabolic disruption.

Lactate derived from LDHA activity in both tumor cells and DCs undermines DC function through multiple interconnected pathways. It directly weakens antigen presentation and suppresses the secretion of pro-inflammatory mediators, including IL-6, IL-12, and TNF-α, thereby enabling tumor immune escape (24, 77). Lactate also blocks lipopolysaccharide-triggered maturation of monocyte-derived DCs and restrains the differentiation of myeloid progenitors into functional antigen-presenting cells (52, 78). Beyond these indirect impacts, lactate disrupts the intrinsic functional homeostasis of DCs and further impairs their anti-tumor competence (79).

### 4.4 TANs

Beyond DCs, TANs exhibit prominent phenotypic and functional heterogeneity within TME. TANs undergo a stepwise differentiation process originating from the transitional TAN-3 phenotype, progressing through intermediate subsets (TAN-0 and IFN-γ-activated TAN-4), and ultimately terminally polarizing into two functionally distinct terminal subtypes: anti-tumor TAN-2 and pro-tumor TAN-1.

TAN-1 is characterized by the secretion of angiogenic factors (e.g. VEGF) and pro-metastatic matrix metalloproteinases (MMPs), which collectively drive immunosuppression and malignant progression. In contrast, TAN-2 possesses a pro-inflammatory phenotype, upregulates neutrophil activation markers, and potentiates anti-tumor immune responses. TAN-3 resembles circulating peripheral neutrophils and serves as a dynamically transitional subset responsive to TME-derived cues. TAN-4 highly expresses IFN-stimulated genes and participates in adaptive immune regulation (80, 81).

Multi-omics analyses have identified a progressive glycolytic shift during TAN differentiation, wherein glycolytic activity continuously increases and peaks in the TAN-1 subtype. LDHA overexpression drives glycolytic hyperactivation and endows TAN-1 with immunosuppressive and tumor-promoting properties. Clinically, elevated LDHA abundance in TAN-1 is strongly correlated with poor clinical prognosis across multiple solid tumors (80). This process is governed by cell-intrinsic LDHA metabolic reprogramming and subsequent TME acidosis-driven immune suppression. LDHA-fueled glycolysis elevates intracellular lactate buildup, which propagates outward to lower extracellular pH and weaken local anti-tumor immune surveillance. Such lactate-rich acidic milieu facilitates extracellular matrix degradation, amplifies pro-inflammatory cascades, and elevates vascular permeability. Moreover, lactate activates the PI3K/AKT/HIF-1α signaling axis to further sustain neutrophil activation. These interactive perturbations disrupt TME immune homeostasis, highlighting the intrinsic functional divergence among TAN subtypes (82). This discovery offers crucial therapeutic targets for addressing tumor immune evasion.

### 4.5 MDSCs

Similar to the metabolic reprogramming observed in TANs, MDSCs also utilize LDHA-driven glycolysis to sustain their immunosuppressive functions. MDSCs abundantly accumulate within TME and markedly constrain the activity of multiple immune subsets, including T cells, B cells, and NK cells. The expansion and functional maturation of MDSCs are controlled by two interconnected layers: cell-intrinsic LDHA metabolic reprogramming and extracellular lactate-mediated biological regulation.

At the intrinsic metabolic level, LDHA sustains glycolytic turnover and redox homeostasis to support MDSC proliferation and immunosuppressive differentiation. Tumor-secreted inducers trigger LDHA-dependent metabolic reprogramming in MDSCs, accompanied by autophagy suppression and upregulated leucine aminopeptidase expression. Such metabolic rewiring facilitates the secretion of G-CSF and granulocyte-macrophage colony-stimulating factor (GM-CSF). These cytokines collectively modulate MDSC recruitment and activation, promote IL-10 secretion, and ultimately reinforce the immunosuppressive TME (83–85).

Excess lactate accumulated in the TME further strengthens MDSC expansion and enhances their inhibitory potency. Metabolite exposure restrains the proliferation of CD4^+^ and CD8^+^ T cells, stabilizes the suppressive function of Treg cells, and weakens the cytotoxic capability of NK cells (52, 86, 87).

### 4.6 MCs

MCs exert context-dependent dual roles in tumor progression, functioning as either malignant promoters or tumor suppressors, and their biological behaviors are tightly modulated by TME (88). LDHA-mediated metabolic reprogramming dominates the functional regulation of MCs, acting mainly through cell-intrinsic metabolic rearrangement and extracellular lactate-mediated biological modulation. Elevated LDHA expression enhances glycolytic flux and increases lactate and ATP production. Accumulated lactate further modulates MC activity via specific receptors, including MAS-related G protein-coupled receptor X2 (MRGPRX2), which restrains early MC activation and calcium-dependent degranulation. Consequently, MCs secrete reduced levels of pro-tumor cytokines (e.g., CCL2, IL-6, and TNF-α), thereby diminishing angiogenesis and tumor proliferation (89, 90). Furthermore, LDHA-mediated metabolic rewiring suppresses nuclear factor-kappa B (NF-κB) nuclear translocation and blunts MC-driven inflammatory responses (91, 92).

The lactate-abundant chronic inflammatory TME further remodels MC behaviors via HIF-1α-mediated downregulation of microRNA-155-5p (miR-155-5p). This molecular alteration inhibits IL-33-triggered cytokine secretion (93, 94). Such cytokine regulatory crosstalk between MCs and macrophages potentially mediates anti-tumor immune responses.

## 5 Discussion and perspective

### 5.1 Cell type-specific regulatory mechanisms of LDHA/lactate in tumor immunity

The immunomodulatory functions of LDHA/lactate in tumor immunity are neither uniform nor ubiquitous. Instead, these biological behaviors are tightly determined by the intrinsic metabolic characteristics of immune cells, the expression patterns of MCTs and lactate receptors, and the unique physicochemical properties of the acidic TME (**Tables 1**, **2**). First, distinct immune subsets exhibit intrinsically distinct metabolic dependencies. Pro-inflammatory M1 macrophages rely on robust glycolysis and produce abundant lactate, whereas anti-inflammatory M2 macrophages predominantly depend on mitochondrial OXPHOS and highly express MCTs to efficiently uptake exogenous lactate. Within the TME, internalized lactate is converted into pyruvate and subsequently shuttled into the TCA cycle in M2 macrophages. This metabolite not only serves as an energy fuel but also facilitates M2-like polarization and consolidates immunosuppressive phenotypes.

**Table 1:** Intrinsic LDHA-driven metabolic regulation across tumor-infiltrating immune cells.

Mechanistic category	Mediators & transporters	Mechanistic overview	Immune cell subsets	Immunological impact	Tumor biological outcome
Glycolysis-dependent immune activation	GLUT1, MCT1	Basal LDHA maintains steady glycolysis, supports energy supply and redox homeostasis to sustain the activation, differentiation and effector functions of multiple immune cells. In B cells, LDHA dominates glycolysis under naive status, while metabolic flux declines during plasma cell maturation to facilitate antibody maturation.	M1 macrophages, CD4^+^ Th1, CD8^+^ T, NK cells, Naive B cells/Plasma cells	Enhances phagocytosis, pro-inflammatory cytokine secretion and cytotoxicity; modulates B-cell maturation and humoral immunity	Inhibits tumor growth and immune escape
Immune phenotypic polarization reprogramming	HIF-1α, c-Myc	Excessive LDHA activation drives metabolic reprogramming, facilitating M1-to-M2 transition and balancing Th17/Treg differentiation	M1/M2 macrophages, Th17, Treg cells	Shifts toward immunosuppressive phenotype; elevates FOXP3 stability	Promotes tumor immune tolerance
MDSC-intrinsic immunosuppressive metabolism	G-CSF, GM-CSF	LDHA-dependent glycolysis supports MDSC proliferation and activation, indirectly inhibiting T, B and NK cell immune activity	MDSCs	Broadly suppresses adaptive and innate immune responses	Constructs immunosuppressive TME
Inflammatory metabolic modulation	MRGPRX2, NF-κB	LDHA-driven glycolysis regulates MCs degranulation and NF-κB-mediated inflammatory cascades	MCs	Moderately restrains pro-tumor inflammatory cytokine release	Exerts dual tumor regulatory effects

This table summarizes cell-intrinsic LDHA-dependent metabolic characteristics and corresponding immunological and tumor-regulatory outcomes across major tumor-infiltrating immune cell subsets.

**Table 2:** Extracellular lactate, receptor signaling, acidification and lactylation-mediated immune modulation.

Mechanistic category	Mediators & transporters	Mechanistic overview	Immune cell subsets	Immunological impact	Tumor biological outcome
GPR81/MCT-mediated immune suppression	GPR81, MCT1/4	Extracellular lactate engages GPR81/MCT signaling to dampen antigen presentation, cell maturation and pro-inflammatory responses	DCs, monocytes, Th2/Tfh cells	Blunts the initiation of anti-tumor immune priming	Permits tumor immune evasion
NK cell subtype-specific metabolic inhibition	NKp46	Lactate selectively downregulates NKp46 expression to impair NK cytotoxicity without affecting NKG2D or NKp30 function	NK cells	Reduces cytotoxic granule secretion and anti-tumor killing	Facilitates tumor immune escape
TME acidification-induced immune dysfunction	PH-sensing machinery	Sustained lactic acidosis inhibits T cell activation and chemotaxis, and stabilizes pro-tumor polarization of M2 macrophages and TANs	CD4^+^ T cells, M2 macrophages, TANs	Attenuates adaptive immunity and locks immunosuppressive phenotypes	Accelerates tumor angiogenesis and metastasis
HIF-1α-mediated epigenetic modulation	HIF-1α, ARG1	Lactate-driven HIF-1α activation reinforces the immunosuppressive transcriptional profile of M2 and Treg cells	M2 macrophages, Treg cells	Maintains chronic immune suppression	Sustains pro-tumor microenvironmental homeostasis
AARS1/SIRT3-dependent protein lactylation	AARS1, SIRT3	Lactate serves as a substrate for AARS1-catalyzed protein lactylation; SIRT3-mediated de-lactylation restores mitochondrial and immune function	CD8^+^ T cells, NK cells	Disrupts immune infiltration and checkpoint regulation; rescues cytotoxicity	Modulates tumor immune checkpoint resistance

This table categorizes distinct mechanistic layers of lactate action, including transporter/receptor-mediated immune modulation, microenvironmental acidosis, and AARS1/SIRT3-dependent protein lactylation. These pathways are mechanistically independent and should not be generalized as a single lactate signaling cascade.

Second, lactate-mediated biological effects are highly dependent on the expression abundance of MCTs and G-protein coupled receptor 81 (GPR81). In lung adenocarcinoma, elevated MCT4 expression is positively correlated with Th2 cell infiltration, indicating the superior lactate uptake capability of Th2 cells. Tumor cell-derived lactate is exported via MCT4 and internalized by Th2 cells, which acts as either an energy substrate or a mediator to sustain Th2 polarization and functional stability. Moreover, the heterogeneous expression of MCTs governs the activation responses of CD8^+^ T cells, monocytes, and other immune subsets, thereby determining their differential sensitivity to lactate signaling.

Third, TME physicochemical properties profoundly shape lactate-driven immunomodulation. LDHA-mediated lactate generation and efflux trigger extracellular acidification, and immune cells display divergent acid tolerance: low pH conditions markedly suppress the activity of CD4^+^ and CD8^+^ T cells, whereas an acidic milieu further promotes M2 macrophage polarization and strengthens their immunosuppressive properties. Collectively, these alterations collaboratively construct an immunosuppressive TME. Therapeutically, pharmacological blockade of lactate efflux using MCT inhibitors or hydrogen sulfide (H_2_S)-generating nanotherapeutics effectively reverses immune suppression and restrains tumor progression (95, 96).

Finally, lactate-mediated regulation of immune cell fate is tightly coupled to intrinsic transcriptional signatures. Specifically, the expression level of HIF-1α modulates the differentiation balance of pivotal immune subsets, including Th17/Treg and M1/M2 phenotypes, thereby remodeling the immune landscape of TME. Endogenously produced lactate within activated T cells sustains metabolic fitness and facilitates effector differentiation, while supraphysiological exogenous lactate derived from tumor cells exerts potent immunosuppressive effects. Collectively, the immunomodulatory actions of LDHA and lactate are neither invariable nor uniform. These biological behaviors are jointly determined by immune cell subtypes, metabolic dependencies, transporter and receptor expression profiles, the degree of TME acidification, and downstream transcriptional cascades, thereby constructing an intricate, cell-specific, context-dependent regulatory network.

### 5.2 Research challenges and directions of LDHA inhibitors

LDHA possesses profound translational clinical value and serves as a reliable biomarker for tumor prognostic assessment, therapeutic response monitoring, and patient stratification. Tissue-derived LDHA acts as a specific mechanistic and therapeutic target, whereas serum LDH serves only as a non-specific auxiliary prognostic biomarker. Serum LDH elevation may reflect not only tumor glycolytic activity but also general tumor burden, tissue injury, systemic inflammation, and widespread metabolic stress, thereby lacking strict tumor specificity and carrying inherent limitations for precise patient stratification (97, 98). Accumulating clinical evidence indicates that abnormal serum LDH levels are closely correlated with patient survival. For example, elevated LDH is strongly associated with poor clinical outcomes in breast cancer patients with bone metastasis (99). In triple-negative breast cancer (TNBC), elevated preoperative serum levels of LDH and alkaline phosphatase (ALP) are tightly associated with shortened disease-free survival (DFS) and overall survival (OS). Moreover, the combined upregulation of these two serum biomarkers efficiently identifies TNBC patients with dismal clinical prognosis (100). Currently, clinical investigations focusing on LDHA and lactate metabolism are progressively advanced. Multiple clinical trials have adopted LDHA expression or serum LDH activity as core indicators for prognostic stratification and therapeutic efficacy evaluation. For instance, the ClinicalTrials.gov-registered trial NCT01878422 provides compelling clinical evidence supporting the feasibility of LDHA as a actionable prognostic biomarker (101, 102).

Against this clinical backdrop, a panel of synthetic LDHA inhibitors with diverse chemical scaffolds has exhibited potent anti-tumor efficacy in preclinical models, including N-hydroxyindoles, gossypol derivatives (e.g., oxamate), quinoline carboxylic acid-based FX-11, and FK866 (103–108). Besides small-molecule agents, LDHA small interfering RNA (siRNA) nanotherapeutics have also been reported to suppress M2 macrophage polarization and enhance autophagic activity, thereby improving oxaliplatin sensitivity in colorectal cancer (109). Although these LDHA-targeted agents exert robust anti-tumor activities in basic research and achieve synergistic anti-cancer effects with immunotherapy, they confront substantial translational bottlenecks. To date, no LDHA inhibitor has been officially approved for clinical application, and most candidate compounds remain in the virtual screening or preclinical validation stage. Overall, the clinical translation of current LDHA-targeted strategies is predominantly constrained by three core limitations: intrinsic structural defects of candidate molecules, tumor metabolic heterogeneity, and systemic toxic side effects.

Conventional LDHA inhibitors exhibit prominent structural drawbacks. Most reported small-molecule inhibitors (represented by GNE-140) rely on polar functional groups (e.g., hydroxyl and carboxyl groups) to form hydrogen bonds with His192 and Arg168 residues within the LDHA catalytic pocket, thereby mediating enzymatic inhibition. Nevertheless, such conserved binding patterns severely limit scaffold diversification, resulting in poor isoform selectivity and high off-target toxicity. Meanwhile, the complex amino acid composition and heterogeneous physicochemical properties of the LDHA catalytic groove further hinder the rational design of highly selective inhibitors. Furthermore, dynamic post-translational modifications profoundly reshape the spatial conformation of LDHA. For example, cysteine palmitoylation deficiency restrains pancreatic cancer progression (110, 111), whereas lysine acetyltransferase 7-mediated acetylation activates LDHA and facilitates the metastasis of head and neck squamous cell carcinoma (17). Certain regulatory proteins also stabilize LDHA to accelerate TNBC progression (112). These tissue-specific modification signatures substantially increase the difficulty of developing broad-spectrum LDHA inhibitors. Tumor-intrinsic metabolic plasticity constitutes another critical factor compromising the therapeutic efficacy of LDHA inhibition. Upon glycolytic blockade, tumor cells dynamically activate alternative metabolic pathways to compensate for energy deficiency, including the pentose phosphate pathway, mitochondrial oxidative phosphorylation, and glutaminolysis. Emerging evidence has demonstrated that LDHA inhibition remodels intracellular metabolism via the pyruvate/oxaloacetate/adenosine monophosphate-activated protein kinase (AMPK) axis, enhances NADPH-dependent ferroptosis resistance, and even accelerates the malignant progression of cervical cancer (113). Such context-dependent dual regulatory effects lead to inconsistent therapeutic responses across distinct tumor types, TME conditions, and metabolite concentrations, thereby reducing the controllability of LDHA-targeted therapy. In addition, LDHA is constitutively expressed in normal tissues, including erythrocytes, skeletal muscle, and intestinal epithelium. Non-specific LDHA inhibition disrupts lactate homeostasis in healthy tissues, induces systemic metabolic toxicity, and compresses the clinical therapeutic index (37, 114). Machine learning-assisted screening eliminates the reliance on conventional polar functional groups and facilitates the discovery of unprecedented chemical scaffolds. Specifically, N-phenylbenzenesulfonamide is identified as a novel LDHA inhibitory skeleton through deep reinforcement learning-based iterative screening. Subsequent molecular dynamics simulation, biological activity validation, and structural optimization further generate optimized lead compounds with potent *in vivo* anti-tumor potency. This innovative drug development paradigm provides novel insights for improving inhibitor selectivity and optimizing pharmacokinetic characteristics (115). In summary, LDHA-targeted therapy still faces multiple translational barriers, including monotonous chemical scaffolds and insufficient selectivity of traditional small molecules, metabolic compensation-mediated therapeutic instability, and non-specific toxicity-induced narrow therapeutic index. Multi-dimensional optimization strategies are urgently required to accelerate the clinical translation and practical application of LDHA-targeted anti-tumor regimens.

### 5.3 Targeting LDHA to improve the efficacy of tumor immunotherapy

The combination of LDHA inhibitors and immunotherapy represents a promising therapeutic modality for malignant tumors. LDHA inhibition remodels the metabolic tumor microenvironment and reverses immunosuppression, thereby potentiating the activation and cytotoxicity of chimeric antigen receptor T (CAR-T) cells. Oxamate exerts robust anti-tumor activities through such synergistic mechanisms (116, 117). *In vivo* xenograft tumor models have validated that LDHA knockdown markedly restricts tumor growth and generates synergistic anti-cancer effects when combined with anti-human prostate-specific membrane antigen (hPSMA)-targeted CAR-T cells. Nevertheless, durable tumor eradication remains unattainable due to insufficient xenogeneic CAR-T activity and intrinsic tumor antigen loss (118). In terms of immune checkpoint inhibitor (ICI) therapy, LDHA modulates the expression of mismatch repair (MMR) proteins and determines the differential status of deficient mismatch repair (dMMR) and proficient mismatch repair (pMMR). LDHA inhibitors significantly sensitize pMMR colorectal cancer to PD-1 antibody immunotherapy (119). In bladder cancer, elevated LDHA expression facilitates lactate accumulation, induces E1A binding protein p300 (EP300)-mediated histone H4 lysine 5 (H4K5) lactylation, and directly activates PD-L1 transcription. Conversely, the glycolytic inhibitor 2-deoxy-D-glucose (2-DG) efficiently reverses this immune evasion phenotype (120). Additionally, LDHA targeting improves ICI responsiveness in ICI-resistant renal cell carcinoma (121). Collectively, LDHA inhibitors act as potent immune sensitizers to amplify anti-tumor immune responses and overcome immunotherapeutic resistance, conferring broad translational prospects. Nevertheless, constrained by the intrinsic drawbacks of current LDHA inhibitors, this combinatorial strategy encounters substantial clinical translational challenges. At present, relevant preclinical studies are limited to *in vitro* cellular experiments and animal models, and the clinical efficacy, biosafety, and optimal administration regimens in human patients require further systematic validation (122).

## 6 Conclusion

LDHA is a critical glycolytic enzyme responsible for remodeling the tumor immune microenvironment. LDHA-driven glycolysis and lactate accumulation induce metabolic reprogramming in both tumor cells and immune subsets. The immunomodulatory behaviors of LDHA and lactate are not static but are collectively determined by immune cell subtypes, metabolic dependencies, transporter-receptor profiles, and TME acidification levels. LDHA inhibition effectively reverses metabolic immunosuppression, sensitizes tumors to CAR-T therapy and immune checkpoint blockade, and mitigates immunotherapeutic resistance. However, the clinical application of LDHA-targeted strategies is restricted by low inhibitor selectivity, tumor metabolic compensation, and systemic toxicity. Future studies should prioritize the fabrication of high-selectivity inhibitors and optimized combinatorial schedules. Advanced delivery systems and multi-omics strategies are required to decipher the intricate LDHA-associated immunometabolic network.
